# Erythroferrone-Driven Regulation of Hepcidin and Iron Levels in Polytransfused Sickle Cell Anaemia Patients: A Prospective Study

**DOI:** 10.1155/bmri/6803051

**Published:** 2025-03-26

**Authors:** Samuel Kwasi Appiah, Charles Nkansah, Godfred Amoah Appiah, Gabriel Abbam, Felix Osei-Boakye, Samira Daud, Kofi Mensah, Safo Adwoa, Korah Seedolf Kuwaahsuore, Emmanuel Yeboah, Abu Siraj Salma Tiyumba, Dennis Thompson, Viel Mary Paula, Louis Adda Duibajia, Peter Takyia, Franklina Ataa Kwarteng, Obed Odame Asiedu, Firdaus Ibrahim Sukasorr, Vincent Kawuribi, Boniface Nwofoke Ukwah, Ejike Felix Chukwurah

**Affiliations:** ^1^Department of Haematology, School of Allied Health Sciences, University for Development Studies, Tamale, Ghana; ^2^Department of Medical Laboratory Science, Faculty of Health Science and Technology, Ebonyi State University, Abakaliki, Nigeria; ^3^Department of Biomedical Laboratory Sciences, School of Allied Health Sciences, University for Development Studies, Tamale, Ghana; ^4^Department of Medical Laboratory Technology, Faculty of Applied Science and Technology, Sunyani Technical University, Sunyani, Ghana

**Keywords:** erythroferrone, ferroportin, hepcidin, polytransfused patients, sickle cell anaemia, vaso-occlusive crises

## Abstract

The interplay of erythroferrone (ERFE), hepcidin, and ferroportin is crucial for ensuring systemic iron homeostasis. This study determined the influence of ERFE on hepcidin and iron levels in polytransfused patients with sickle cell anaemia (SCA). This multicentre case-control study recruited 60 SCA participants and 30 controls (HbA), aged 2–34 years, from Tamale Teaching Hospital; Methodist Hospital, Wenchi; and Seventh Day Adventist Hospital, Sunyani, Ghana, between the periods of March to July 2023. About 4 mL of blood was collected for a full blood count using a haematology analyzer and serum ERFE, hepcidin, ferroportin, and ferritin estimation using an enzyme-linked immunosorbent assay. Data were analyzed using SPSS Version 26.0. ERFE (*p* < 0.001), ferroportin (*p* = 0.016), ferritin (*p* < 0.001), serum iron (*p* < 0.001), transferrin (*p* = 0.001), soluble transferrin receptor (sTFR) (*p* = 0.019), TWBC (*p* < 0.001), and platelet (*p* < 0.001) were significantly higher in SCA participants and hydroxyurea-naïve participants than in the control group and hydroxyurea-treated participants, respectively. Levels of hepcidin (*p* < 0.001), red blood cell (*p* < 0.001), haemoglobin (*p* < 0.001), and haematocrit (*p* < 0.001) were lower in the SCA and hydroxyurea-naïve groups than in the control and hydroxyurea-treated groups, respectively. An inverse correlation was observed between serum ERFE and hepcidin (*r* = −0.391, *p* = 0.002) and hepcidin and ferroportin (*r* = −0.266, *p* = 0.040), while ferritin (*r* = 0.439, *p* < 0.001) and ferroportin (*r* = 0.309, *p* = 0.016) showed a positive correlation with ERFE. No correlation was found between serum hepcidin and ferritin levels (*r* = 0.025, *p* = 0.853). Again, participants with regular blood transfusions had significantly higher levels of ERFE (*p* < 0.001) and ferritin (*p* = 0.002) than those with rare and no transfusions per year. None of the SCA participants had done iron testing. In conclusion, the negative impact of ERFE on hepcidin levels may exacerbate the risk of iron burden, as evident by elevated iron levels in SCA patients and the need for regular monitoring of the iron status of polytransfused SCA patients.

## 1. Introduction

Sickle cell anaemia (SCA) is an autosomal recessive genetic disorder that results from a point mutation in the *β*-globin gene and affects red blood cells, leading to chronic anaemia, pain, and organ damage [1, 2]. In SCA, abnormal haemoglobin (Hb) creates sickle-shaped red blood cells at low oxygen tension, which can obstruct blood arteries, reducing oxygen supply to the tissues [3]. Globally, approximately 300,000 people are born each year with sickle cell disease (SCD), with 80% occurring in Africa [3]. In Ghana, about 2.0% of newborns have SCD, of which 55% have SCA [4].

Management of sickle cell patients with transfusions improves blood circulation and transport of oxygen by reducing the percentage of red blood cells that can form sickle Hb polymers, which prevents the associated haemolysis and endothelial damage [5, 6].

The episodic and chronic nature of transfusion therapy in patients with SCA causes an excessive build-up of iron in the body and leads to iron overload that may go undetected. The excessive accumulation of iron in the body generates abundant reactive oxygen species, which may be toxic and have detrimental effects on visceral organs, especially the liver and heart [7].

There is no distinct physiological mechanism to excrete excess iron from the body; hence, the regulation of iron stores is entirely dependent on changes in iron absorption through the hepcidin-ferroportin axis [8]. The hormone erythroferrone (ERFE) is an erythroid regulator of hepcidin and iron homeostasis in response to erythropoietin stimulation caused by haemorrhage, hypoxia, and inherited Hb disorders such as SCA [9]. ERFE controls iron metabolism by suppressing the hepatic expression of the iron-regulator hormone hepcidin, mediated by interference with the action of the bone morphogenetic protein signalling pathway that regulates iron transcription [10]. Hepcidin is the primary regulator of iron absorption in humans, and its deficiency impairs the ability of the body to regulate the entry of iron mediated by ferroportin in response to iron overload, erythropoiesis, and inflammation [11]. Ferroportin is a protein that helps several cell types, mainly enterocytes and macrophages of the reticuloendothelial system, to export iron [12]. Hepcidin forms a complex with ferroportin, and this complex is absorbed by these cells, where it causes ferroportin to degrade, inhibiting iron outflow, and as a result, reduces intestinal iron absorption and bioavailability [13].

ERFE has been suggested to play a possible role in SCA pathogenesis. Previous studies reported elevated levels of ERFE in SCA patients compared to healthy controls, and the protein correlated positively with markers of haemolysis and inflammation [14, 15]. Chronic haemolysis and persistent upregulation of ferroportin have been implicated in the pathogenesis of vaso-occlusion and pain crises experienced in SCA [16].

Blood transfusion, in addition to increased absorption of iron from the gut by the interaction of the ERFE-hepcidin and ferroportin axis, contributes to increased availability of iron, thereby predisposing SCA patients to iron burden [11].

A study by Kautz et al. [17] demonstrated that ERFE is a major regulator of hepcidin during erythropoietic stress, iron deficiency, and anaemias, providing foundational insights into its role in iron homeostasis. Elevated levels of hepcidin are generally seen in anaemia of chronic disease or inflammation, where cytokines (such as IL-6) promote its synthesis. In contrast, ERFE directly suppresses hepcidin production in order to facilitate iron mobilization, which is particularly relevant in SCA patients, who may suffer from both anaemia and increased iron demand due to chronic blood destruction [18, 19]. The inverse correlation between ERFE and hepcidin observed in SCA is consistent with the understanding that ERFE suppresses hepcidin to ensure adequate iron availability for erythropoiesis. This finding parallels previous work in SCA and related conditions in the United Arab Emirates [20].

More recently, studies like Mangaonkar et al. [21] have investigated the role of ERFE in SCA, showing that polytransfused patients exhibit elevated ERFE levels, which contribute to exacerbated iron overload.

Currently, data on ERFE, hepcidin, and ferroportin in SCA in Ghana is limited, and therefore, there is a need to assess serum levels of ERFE among polytransfused SCA patients and its relationship with hepcidin and ferroportin. The study findings will be essential in the clinical management of SCA.

## 2. Methodology

### 2.1. Study Design and Population

This multicenter study was conducted at the Tamale Teaching Hospital (TTH); Methodist Hospital, Wenchi; and Seventh Day Adventist (SDA) Hospital between the periods of March to July 2023. TTH serves residents of the five northern regions and is a tertiary-level referral facility situated in Tamale, the capital of the northern region, with a total population of 374,766 and a digital address of NT-0101-5777 [22]. Wenchi Methodist Hospital is a 250-bed facility that serves as a referral center for 20 healthcare facilities, both government and private, in the municipality with a population of 124,758 and a digital address of BW-0005-0306 [23]. The SDA Hospital, located at 7°20⁣′ N 2°20⁣′ W, is the region's first-level referral hospital, offering general and specialized medical care to the 208,496 residents of the Bono region's capital city [24]. The study recruited a total of 90 participants (60 participants with SCA and 30 healthy controls (HbA), aged 2–34 years, who attended the outpatient clinic of the selected centers.

The study received approval from the Institutional Review Board (IRB) of the University for Development Studies (UDS), Tamale, Ghana (UDS/RB/006/23). Permission was sought from the management of the TTH, Methodist Hospital; Wenchi; and SDA Hospital. Written informed consent was obtained from the participants or their guardians (minors below 18 years of age).

Kelsey's formula for case-control studies was used to estimate the sample size for the study. Participants with comorbidities such as diabetes mellitus, hypertension, human immunodeficiency virus (HIV), hepatitis, pregnant women, and lactating mothers were excluded from this study. Sociodemographic and clinical information were obtained from clinical records. Severity scores were calculated based on the patient's clinical data, as proposed earlier by Hedo et al. [25]. The total score was classified as mild SCA (score < 3), moderate SCA (score > 3 and ≤ 7), and severe SCA (score > 7) [26].

### 2.2. Laboratory Procedures

Four milliliters of venous blood samples from each patient was aseptically phlebotomised into ethylenediaminetetraacetic acid (EDTA) (3 mL) and serum separator tubes (2 mL). The EDTA whole blood samples were used for the analysis of the full blood count using an automated five-part haematology analyser (Sysmex XN-550, Japan), a sickle slide test using sodium metabisulphite, and Hb variants determined using the cellulose acetate electrophoresis method at an alkaline pH of 8.4. The gel tube sample was allowed to clot and spun at 3500 rpm for 5 min. The resulting serum was transferred into Eppendorf tubes and stored at −70°C until analysis of ERFE, hepcidin, ferroportin, and iron profile (ferritin, serum iron, transferrin, and receptors) using commercially prepared enzyme-linked immunosorbent assay (ELISA) kits (Biobase, China). Microtiter plate wells were coated with specific monoclonal antibodies, forming a solid-phase antibody. Patients/control sera were added to the wells and incubated, and upon the addition of horseradish peroxidase (HRP)–labeled antibody (conjugate), an antibody–antigen–enzyme–labeled antibody complex was formed. After washing using the WHYM201 microplate washer and the addition of TMB substrate solution, a blue-coloured product was formed. A weak sulphuric acid solution was added to terminate the reaction, and the optical density of the resulting colour change was measured using an ELISA reader (Powean-Medical, China) at 450 nm. All the analyses were conducted at the TTH. Already assayed clinical samples were used as internal quality control measures to validate the kits.

### 2.3. Statistical Analysis

Data were analysed using IBM SPSS software Version 26.0 (Armonk, NY, United States). After the normality test, normally distributed data were presented as mean ± standard deviation (SD), and skewed data were presented as median (interquartile range (IQR)). Student's *T*-test and Mann–Whitney *U*-test were used to compare parametric and nonparametric data, respectively. One-way ANOVA or the Kruskal–Wallis test was used to compare continuous data among participants with regular, rare, and no blood transfusion. Statistical significance was set at *p* < 0.05.

## 3. Results

### 3.1. Demographic and Clinical Characteristics of the Study Participants

Of the 90 participants recruited into the study, 60 (66.7%) were SCA patients and 30 (33.3%) were apparently healthy individuals. The median age of the participants was 16.0 years (2.0–34.0). Forty-one (45.6%) of the participants were males, 42 (70%) of cases had visited the hospital between one and three times per year, 38 (63.3%) had experienced vaso-occlusive crises between one and three times per year, and 25 (41.7%) had received one or two blood transfusions in months. In terms of severity score, 34 (56.7%) of the patients were mild, 22 (36.7%) were moderate, and four (6.6%) were severe. None of the participants had previously undergone iron testing (Table 1).

### 3.2. Haematological Parameters of Study Participants

There was a significant difference in haematological parameters between SCA participants and the controls: ERFE (*p* < 0.001), ferroportin (*p* = 0.016), hepcidin (*p* < 0.001), ferritin (*p* < 0.001), serum iron (*p* < 0.001), transferrin (*p* = 0.001), sTFR (*p* = 0.019), and TSAT% (*p* = 0.001) were significantly higher in the participants with SCA than those in the control group.

On the other hand, sickle cell anaemic participants had lower RBC count (*p* < 0.001), Hb (*p* < 0.001), and haematocrit (HCT%) (*p* < 0.001) but higher mean cell volume (MCV) (*p* < 0.001), RDW-CV% (*p* < 0.001), total white blood cell (TWBC) (*p* < 0.001), and platelet (*p* < 0.001) (Table 2).

### 3.3. Haematological Parameters of Study Participants Stratified by Hydroxyurea Usage

Sickle cell anaemic participants on hydroxyurea therapy had significantly increased hepcidin (*p* = 0.012), RBC (*p* = 0.001), Hb (*p* < 0.001), and HCT (*p* = 0.031) compared with hydroxyurea-naïve patients. However, serum ERFE (*p* < 0.001), ferroportin (*p* = 0.002), ferritin (*p* < 0.001), serum iron (*p* = 0.014), sTFR (*p* = 0.001), transferrin (*p* < 0.001), MCV (*p* = 0.014), TWBC (*p* < 0.001), and platelet (*p* < 0.001) were relatively reduced in the SCA patients on hydroxyurea therapy than those who had not received hydroxyurea (Table 3).

### 3.4. Association Between Serum ERFE, Hepcidin, and Ferritin Levels in SCA Participants

**Figure 1:**
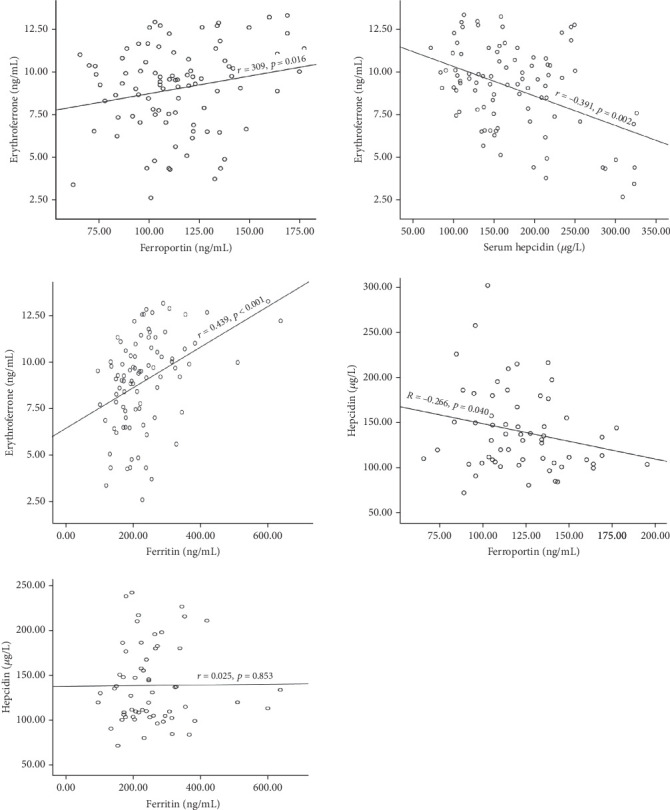
Association between serum erythroferrone, hepcidin, and ferritin in SCA participants. *r* = correlation coefficient, *μ*g/L = microgram per liter, ng/mL = nanogram per milliliter. Spearman's correlation test was used to determine the correlation between the parameters. *p* < 0.05 was considered statistically significant.

There was an inverse correlation between serum levels of ERFE and hepcidin (*r* = −0.391, *p* = 0.002) and hepcidin and ferroportin (*r* = −0.266, *p* = 0.040). Ferritin (*r* = 0.439, *p* < 0.001) and ferroportin (*r* = 0.309, *p* = 0.016), however, showed a positive correlation with ERFE. Hepcidin and ferritin showed no relation (*r* = −0.214, *p* = 0.043) (Figure 1).

### 3.5. Ferritin and Iron Regulatory Markers Among Study Participants Stratified by Frequency of Transfusion per Year

Participants who received regular blood transfusions had significantly higher levels of ERFE (*p* < 0.001) and ferritin (*p* = 0.002) than those with rare and no transfusions per year. The levels of hepcidin were found to be lower in participants with regular transfusion compared to the group with no or rarely received blood transfusion (Table 4).

## 4. Discussion

Inappropriate expression of ERFE suppresses hepatic synthesis of hepcidin, and this could contribute to the pathogenesis of iron overload in polytransfused SCA patients. This study determined the influence of ERFE on the levels of hepcidin, ferroportin, and iron profile in patients with SCA in Ghana. The median age of the study participants was 16.0 years (2.0–34.0), with the majority of the SCA participants hospitalized at least once per year (86.7%), one blood transfusion per year (71.7%), and one episode of vaso-occlusive crisis per year (90%). The high occurrence of vaso-occlusive crises leading to hospitalization was a significant contributory factor necessitating blood transfusions in participants with SCA [27, 28]. In the present study, SCA participants were found to have significantly higher levels of ERFE, ferroportin, ferritin, serum iron, transferrin, sTFR, and TSAT than the control group. The high ERFE and ferroportin observed could be attributed to the persistent stimulation of erythropoiesis triggered by stress anaemia. In cases of ineffective erythropoiesis, an augmented population of erythroblasts produces excess ERFE, which suppresses hepcidin expression and contributes to iron burden, even in nontransfused patients [29, 30]. Previous studies have explored ERFE levels in various forms of anaemia, including SCA. In a study by Kautz et al. [17], ERFE levels were shown to be higher in response to erythropoiesis stimulation in anaemia but with a clear distinction based on the type of anaemia. For instance, in iron deficiency anaemia, ERFE levels were higher compared to healthy controls, which aligns with our findings in SCA, where the body responds to anaemia with an increase in ERFE to facilitate iron mobilization. Again, Talawy et al. [20] reported elevated levels of ERFE in *β*-thalassaemia patients, reflecting the need for enhanced iron mobilization and supply to the bone marrow to enhance effective erythropoiesis. These findings support the hypothesis that ERFE acts as a compensatory response to stress and chronic anaemias, emphasizing the relevance of the current findings in SCA.

However, a key difference in SCA, compared to other anaemias like iron deficiency anaemia or *β*-thalassaemia, is the chronic nature of haemolysis. In contrast to conditions where iron is generally lacking, SCA patients often experience a paradoxical state of iron overload due to the increased destruction of red blood cells and subsequent blood transfusions. Alkindi et al. [7] and Mangaonkar et al. [21] highlighted that in SCA, while ERFE increases to support erythropoiesis, the body faces a dilemma of iron dysregulation, with some tissues like bone marrow being iron-deficient while others like the liver and heart may suffer iron toxicity. This complex iron metabolism in SCA patients is in stark contrast to the relatively more straightforward iron deficiency or thalassaemic conditions studied in other literatures.

Again, the elevated iron profile is in consonance with studies conducted in Congo [31], Saudi Arabia [32], and India [33]. This could probably be due to the episodic chronic blood transfusion and the inhibitory effect of ERFE on a major iron regulatory protein called hepcidin [9, 14]. However, the findings of this study contradict an earlier study conducted in Nigeria that reported lower levels of serum iron and transferrin with normal levels of TIBC and ferritin [34]. The variation in the findings may be attributed to demographics and differences of the study patients. This study recruited steady-state SCA patients aged between 2 and 34 years, whereas the study by Olaniyi recruited patients both in steady-state and in vaso-occlusive crisis, aged between 18 and 40 years.

This study reported significantly lower hepcidin levels in SCA patients than in the healthy control group. Previous studies have reported similar findings [35, 36], and this could be explained by the overriding effect of intense erythropoiesis or hypoxia that downregulates hepcidin synthesis against the stimulatory effect of inflammatory cytokines (IL-6) on hepcidin experienced by SCA patients [37, 38]. This contradicts earlier studies in Egypt [39] and the United States [40] that reported elevated serum hepcidin levels in SCD patients with multiple blood transfusions. The difference in the findings may be due to the variations in the study participants. The earlier studies included *β*-thalassaemia major patients who were transfusion-dependent, while the current study recruited only SCA patients.

Haematological indicators exhibit variability, with significant reductions observed in levels of red blood cell count, Hb, and HCT and elevated RDW-CV, MCV, TWBC, and platelet counts among SCA participants compared to the control group. The decreased levels of HCT, RBC, and Hb observed in our study could be attributed to chronic haemolysis, reduced red cell survival, and a diminished response of erythropoietin related to SCA [41, 42], whereas the high MCV and RDW-CV are consistent with poikilocytosis and reticulocytosis as a consequence of the bone marrow compensatory mechanism in response to the anaemia stress.

The significant leukocytosis observed in the SCA group could be explained by the persistent, subclinical inflammation that results in cytokine release and boosts bone marrow leucocyte production or the functional hyposplenia and infections associated with SCD [43]. The high platelet counts seen among patients with SCA could be a reactive immune response to the inflammation associated with endothelial damage and activation [1]. Also, the thrombocytosis seen in the patients could probably be due to functional hyposplenia, and this is supported by previous studies [44, 45].

The present study found a significant reduction in the levels of ERFE, ferroportin, leucocytes, platelets, and iron levels (ferritin, serum iron, sTFR, and transferrin) with significantly improved red cell indices (Hb, HCT, and RBC) in SCA participants on HU therapy compared to the HU-naïve participants. The improved red cell indices are consistent with previous studies that reported HU to induce foetal haemoglobin (HbF) production, minimize the proportion of HbS, and consequently reduce the incidence of haemolysis, thereby improving the haemogram levels [46–49]. The reduction in leucocyte and platelet can be attributed to the cytoreductive properties of HU, which inhibits DNA synthesis, consequently leading to diminished marrow production of reticulocytes and WBC (neutrophils) that minimizes intracellular sickling, endothelial activation, the rate of haemolysis, and vaso-occlusive crisis [33, 50]. Hydroxyurea therapy also reduces the dependence on blood transfusions in SCA, improves anaemia, and inhibits the effect of ERFE on hepcidin (major iron regulator) production, thereby minimizing the risk of transfusion iron burden [47, 51]. The observed outcome highlights the therapeutic benefit of HU therapy as it minimizes the haematological derangements and clinical symptoms with improvement in the quality of life of individuals with SCA [52].

The correlation between serum ERFE, hepcidin, ferroportin, and ferritin was assessed among study participants. The study observed a significant inverse correlation between serum ERFE and hepcidin, whereas ferritin and ferroportin correlated positively with serum ERFE. Previous studies have reported inappropriate increased expression of ERFE in conditions associated with significant ineffective erythropoiesis, such as SCA, leading to the suppression of hepcidin synthesis [36]. This promotes ferroportin expression, which mediates the release of iron stored in the gut, spleen, and liver by ferroportin into the blood plasma, thus contributing to the worsening of iron overload [11, 14, 20]. The inverse correlation between ERFE and hepcidin in this study is inconsistent with previous studies [53]. The variations in the findings may be related to the differences in the study participants and geographical locations. The previous studies recruited Turkish children with iron deficiency anaemia, while the current study recruited SCA patients. These findings highlight the overriding effect of ineffective erythropoiesis on hepcidin expression independent of associated inflammation or possible iron repletion.

The significant positive correlation observed between ERFE and ferritin highlights the inhibitory effect of ERFE on hepcidin expression, promoting the increased release of iron from storage sites, consequently increasing serum ferritin levels in the bloodstream [30]. This phenomenon is further supported in individuals with iron deficiency anaemia, emphasizing the regulatory role of ERFE in iron metabolism [53].

The positive association between serum ERFE and ferroportin is attributed to ERFE's dual action of hepcidin suppression and increased ferroportin availability, facilitating an increase in iron export from cells [38]. This relationship is of importance in conditions characterized by increased erythropoietic demand.

The observed significant inverse correlation between hepcidin and ferroportin as well as ferritin can be explained through the mechanism of hepcidin binding to ferroportin, resulting in the internalization and subsequent degradation of ferroportin. This process leads to the inhibition of iron transport by ferroportin. The identified relationship, where an elevation in hepcidin levels corresponds to a reduction in ferroportin levels, is pivotal for the maintenance of iron homeostasis within the body. In particular, these findings align with the conclusions drawn by earlier studies [10, 54, 55].

The study also determined the levels of iron regulatory markers in relation to transfusion frequency. Serum ferritin and ERFE levels increased proportionally with the number of blood transfusions received during the last year with decreased levels of hepcidin. The rate of iron loading in participants with regular transfusions coupled with intense erythropoietic stress due to chronic anaemia/hypoxia as a consequence of SCA complication upregulates the expression of ERFE by the erythroblast. This suppresses hepatic expression of hepcidin and disables control of ferroportin-mediated influx of iron into the body [31, 32, 56]. The findings from this present study contradict a previous study that reported higher levels of hepcidin in *β*-thalassaemia major patients with the justification that regular transfusions inhibit erythropoietic drive that leads to an increased level of hepcidin [39]. The study is limited by the inability to perform hepatic imaging as the confirmatory test for diagnosing iron overload among the study participants with a higher risk of iron burden.

## 5. Conclusion

The study underscores the impact of ineffective erythropoiesis on excess ERFE production, hepcidin suppression, and consequent iron overload, even in nontransfused SCA patients. Future research is recommended to further elucidate the possible prognostic impact of serum ERFE in the management of SCA patients.

## Figures and Tables

**Table 1:** Demographic and clinical characteristics of the study participants.

**Variables**	**Category**	**Frequency (%)**
Age (years)	16.0 (2.0–34.0)	

Age category	12–20	55 (61.1)
	21–30	30 (33.3)
	> 30	5 (5.6)

Sex	Males	41 (45.6)
	Females	49 (54.4)

Frequency of hospital visits per year	None	8 (13.3)
	1–3	42 (70)
	> 3	10 (16.7)

Number of blood transfusions per year	0	17 (28.3)
	1–2	25 (41.7)
	≥ 3	18 (30.0)

Vaso-occlusive crises per year	None	6 (10)
	1–3	38 (63.3)
	> 3	16 (26.7)

Severity score category	Mild	34 (56.7)
	Moderate	22 (36.7)
	Severe	4 (6.6)

Iron profile test	Yes	0 (0)
	No	60 (100)

*Note:* Categorical data were presented in frequencies with corresponding percentages in parentheses. Age in years was presented as median (25th–75th percentiles.

**Table 2:** Haematological parameters of the study participants.

**Haematological parameters**	**Participants**		**p** ** value**
	**Cases (SCA)** **N** = (60)	**Controls (HbA)** **N** = (30)	
*Iron profile*			
ERFE (ng/mL)	9.9 (8.8–11.5)	7.6 (5.0–9.6)	**< 0.001**
Hepcidin (*μ*g/L)	128.5 (105.2–173.5)	208.6 (150.6–226.6)	**< 0.001**
Ferroportin (ng/mL)	120.3 (105.1–138.5)	110.1 (86.3–119.9)	**0.002**
Ferritin (ng/mL)	240.4 (186.6–309.8)	194.6 (154.7–224.0)	**< 0.001**
Serum iron (mg/dL)	166.7 (123.5–235.8)	99.5 (89.7–112.5)	**< 0.001**
Transferrin (mg/dL)	304.0 (209.3–390.5)	208.7 (162.3–301.4)	**< 0.001**
sTFR (mg/L)	4.5 (3.1–6.0)	3.9 (3.1–4.6)	**0.019**
TSAT%	42.6 ± 17.0	33.2 ± 9.8	**0.010**
*Blood cell parameters*			
RBC ×10^6^/*μ*L	2.6 (2.3–3.4)	4.5 (4.1–4.9)	**< 0.001**
Hb (g/dL)	8.2 (7.1–9.6)	12.7 (12.1–13.9)	**< 0.001**
HCT%	25.6 ± 5.4	39.6 ± 3.8	**< 0.001**
MCV (fL)	88.9 (80.3–97.3)	80.5 (74.7–86.6)	**< 0.001**
RDW-CV%	18.2 ± 3.7	8.3 ± 0.7	**< 0.001**
TWBC ×10^3^/*μ*L	10.3 (8.3–12.6)	5.2 (4.3–5.9)	**< 0.001**
PLT ×10^3^/*μ*L	357.0 (248.5–445.5)	240.0 (180.5–264.5)	**< 0.001**

*Note:* Parametric data presented as mean ± standard deviation was compared using the Student *T*-test, and nonparametric data presented as median (25th–75th) were compared using the Mann–Whitney *U*-test. *p* < 0.05 was deemed statistically significant. *p* values in bold are deemed significant.

Abbreviations: *μ*g/L = microgram per liter, ERFE = erythroferrone, fL = femtoliter, g/dL = gram per deciliter, Hb = haemoglobin concentration, HCT = haematocrit, MCV = mean cell volume, *N* = number of participants, ng/mL = nanogram per milliliter, pg = picogram, PLT = platelet count, RBC = absolute red blood cell count, RDW-CV = red blood cell distribution width-coefficient of variation, SCA = sickle cell anaemia, sTFR = soluble transferrin receptor, TSAT = transferrin saturation, TWBC = total white blood cell count.

**Table 3:** Haematological parameters of study participants stratified by hydroxyurea usage.

**Haematological parameters**	**Participants**		**p** ** value**
	**HU-treated cases (** **N** = 32**)**	**HU-naïve cases (** **N** = 28**)**	
ERFE (ng/mL)	8.6 ± 1.7	11.4 ± 1.3	**< 0.001**
Hepcidin (*μ*g/L)	142.3 (109.2–184.3)	111.7 (101.0–141.3)	**0.010**
Ferroportin (ng/mL)	112.8 ± 21.0	133.2 ± 27.9	**0.002**
Ferritin (ng/mL)	211.1 (175.4–259.8)	360.1 (274.0–525.6)	**< 0.001**
Serum iron (mg/dL)	150.4 (117.5–177.4)	178.7 (144.4–246.9)	**0.014**
sTFR (mg/L)	3.8 (2.9–4.9)	5.7 (4.3–8.1)	**0.001**
Transferrin (mg/dL)	215.0 (177.8–308.5)	375.0 (319.8–442.0)	**< 0.001**
TSAT (%)	42.4 ± 12.9	42.8 ± 21.0	0.921
RBC ×10^6^/*μ*L	3.0 (2.5–4.1)	2.3 (2.0–2.7)	**0.001**
Hb (g/dL)	9.2 ± 1.5	7.5 ± 1.7	**< 0.001**
HCT%	27.0 ± 4.8	24.0 ± 5.6	**0.031**
MCV (fL)	85.3 ± 9.3	94.5 ± 14.1	**0.014**
TWBC ×10^3^/*μ*L	9.1 (7.6–10.9)	12.0 (9.6–14.3)	**< 0.001**
PLT ×10^3^/*μ*L	262.0 (208.3–360.0)	441.0 (357.0–543.5)	**< 0.001**

*Note:* Parametric data presented as mean ± standard deviation was compared using Student's *T*-test, and nonparametric data presented as median (25th–75th) were compared using Mann–Whitney *U*-test. *p* < 0.05 was deemed statistically significant. *p* values in bold are deemed significant.

Abbreviations: % = percentage, *μ*g/L = microgram per liter, *μ*L = microliter, ERFE = erythroferrone, fL = femtoliter, g/dL = gram per deciliter, Hb = haemoglobin concentration, HCT = haematocrit, HU = hydroxyurea, *N* = number, ng/mL = nanogram per milliliter, pg = picrogram, PLT = platelet count, RBC = absolute red blood cell count, sTFR = soluble transferrin receptor, TSAT = transferrin saturation, TWBC = total white blood cell count.

**Table 4:** Ferritin and iron regulatory markers among study participants stratified by frequency of transfusion per year.

**Biochemical parameters**	**Transfusion pattern**			**p** ** value**	**Pairwise comparisons**
	**No transfusion** ^**a**^	**Rare transfusion (1–2 per year)** ^**b**^	**Regular transfusion (≥ 3 per year)** ^**c**^		
ERFE (ng/mL)	8.6 ± 1.7	9.7 ± 2.0	11.5 ± 1.5	< 0.001	a&b, b&c, a&c
Hepcidin (*μ*g/L)	179.2 (114.3–202.6)	128.9 (104.8–149.9)	111.6 (100.8–139.4)	0.046	a&c
Ferroportin (ng/mL)	113.1 ± 20.4	121.5 ± 24.6	132.9 ± 31.4	0.136	NA
Ferritin (ng/mL)	220.1 (153.6–267.1)	211.1 (182.5–276.8)	313.0 (247.3–398.6)	0.002	a&c, b&c

*Note:* Parametric data presented as mean ± standard deviation was compared using one-way ANOVA, and nonparametric data presented as median (IQR = interquartile ranges) were compared using the Kruskal–Wallis test. *p* < 0.05 was deemed statistically significant. a&b, pairwise comparison between no and rarely transfused participants; b&c, pairwise comparison between rarely and regular transfused participants; a&c, pairwise comparison between no and regular transfused participants.

Abbreviations: *μ*g/L = microgram per liter, ng/mL = nanogram per milliliter.

^a^Participants with no transfusion per year.

^b^Rarely transfused participant (1–2 per year).

^c^Regular transfused participants (3 or more per year).

## Data Availability

The data that support the findings of this study are available from the corresponding author upon reasonable request.
